# Asthma

**Published:** 1891-03

**Authors:** L. B. Firth


ASTHMA.—

℞ Fl. ext. grindeliae robustae, ℥ ij.

Potassii iodidi, ʒ ij.

Syr. tolu, q. s. ad., ℥ iv.

M. Sig. A teaspoonful every three hours.

—*Dr. L. B. Firth.*

